# Factors, Attitudes, and Prevalence of Self-Medication Among Pregnant Women: A Cross-Sectional Study in Saudi Arabia

**DOI:** 10.3390/pharmacy13050138

**Published:** 2025-10-01

**Authors:** Alla Alhumaid, Noha Alhumaid, Khalid Alkhurayji, Abdallah Alsuhaimi, Fawaz Modahi, Noor Almanidi, Abdullah Almutairi, Abdullah Alanazi, Nayif Modahi

**Affiliations:** 1 Imam Abdulrhman bin Faisal Hospital, Ministry of Health, Riyadh 14723, Saudi Arabia; aalhumaid4@moh.gov.sa; 2 College of Public Health & Health Informatics, King Saud bin Abdulaziz University for Health Sciences, Riyadh 11481, Saudi Arabia; humaidn@ksau-hs.edu.sa; 3 Research, Statistics, and Information Department, Saudi Central Board for Accreditation of Healthcare Institutions, Riyadh 12264, Saudi Arabia; 4 Executive Department of Standards, Saudi Central Board for Accreditation of Healthcare Institutions, Riyadh 12264, Saudi Arabia; aalsuhaimi@cbahi.gov.sa; 5 Dental Department, King Abdulaziz Medical City, Riyadh 11426, Saudi Arabia; modahifa@mngha.med.sa (F.M.); almanidino@mngha.med.sa (N.A.); almutairiab21@mngha.med.sa (A.A.); alanaziab61@mngha.med.sa (A.A.); modahina@mngha.med.sa (N.M.)

**Keywords:** self-medication, pregnancy, public health, awareness

## Abstract

Background: Self-medication (SM) among women during pregnancy poses a critical risk to maternal health, and SM is still commonly practiced in Saudi Arabia. Therefore, this study aims to assess the factors, attitudes, and prevalence of SM among pregnant women in the Ministry of Health (MOH) First Health Cluster. Methods: A cross-sectional design was used among 400 pregnant women who received care at primary, secondary, and tertiary healthcare levels. A structured tool was adopted and modified based on the literature review, expert and focus group interviews, and the experiences of the target participants. The dependent variables included history of illness, access to healthcare services, medication usage, and perception of SM, while the independent variables included socioeconomic status. Statistical Package for the Social Sciences (SPSS), Version 25 was used for analysis. Results: The prevalence of SM was 36.5%. Cough syrup and antipyretics were the most commonly used medications, while hair problems and weight loss were the most frequently reported reasons for SM. The primary sources of information guiding SM behavior were prior prescriptions and previous experience. Education level, occupation, age, number of pregnancies, and miscarriage history were all significantly associated with SM (*p* < 0.05). Conclusions: SM was found to be frequent among pregnant women in the First Health Cluster, highlighting the need for educational interventions and regulatory measures to reduce unsafe practices and improve maternal health.

## 1. Introduction

Self-medication (SM) is considered a preventable attitude among many people, which is usually driven by over-the-counter (OTC) medication availability, which is considered easy to obtain without appropriate medication prescriptions [1]. This attitude, in particular among women during pregnancy, increases the risk of potential harm to both fetal and maternal well-being [2]. There is a clear empirical research gap regarding SM among pregnant women in certain countries, as previous investigations have not fully explored differences between primigravidae and multigravidae, nor how occupation, education level, clinical characteristics, or cultural factors influence attitudes towards SM [3,4,5,6].

The prevalence of SM among women during pregnancy ranges widely across countries, with some studies reporting rates between 40% and 60% [7,8]. Several mutual factors reported across countries, involving previous experiences with the drugs, ease of access to medications, and perceived mildness of sicknesses, contribute to this high prevalence [9]. However, in Saudi Arabia, access to healthcare services and social culture are considered among the factors that may influence the prevalence of SM [10].

Investigations in the Riyadh region among pregnant women have reported a moderate range of SM practice: between 20–50% [11,12]. These public health concerns warrant significant attention from policymakers and healthcare providers [13]. To illustrate this case, Raheel, Alsakran [14] conducted a study among 354 pregnant women attending antenatal clinics in Riyadh Region and found that approximately 61% reported being unaware of the risks of self-mediation.

This finding is in line with prior investigations, which indicate that the most commonly used drugs for SM among pregnant women include analgesics, antipyretics, antihistamines, antacids, herbal medicines, and nutritional substances. Furthermore, each medication class could carry specific fetal or maternal risks. For example, non-steroidal anti-inflammatory drugs (NSAIDs) could increase the risk of pulmonary hypertension in neonates or precipitate preterm labor. Similarly, the inappropriate use of herbal remedies can increase the chance of unpredictable teratogenic effects. Moreover, misuse of analgesics and antipyretics may cover symptoms of underlying infections, lead to hepatic or renal complications, and delay proper treatment. Awareness of these commonly used medications and the associated risks is considered a crucial part of public health initiatives to develop targeted educational campaigns, counseling during clinical visits, and policy development to prevent unsafe SM attitudes among pregnant women [7,8,9].

Adverse drug reactions, incorrect dosing, and interactions with other medications could happen due to SM [15]. These hazardous practices, particularly during pregnancy, could be exacerbated by physiological changes that could affect drug excretion and metabolism [16]. In addition there are associated risks to fetal development due to the potential teratogenic effects of certain drugs [17]. Nonetheless, SM may result in exposure to certain harmful substances during pregnancy and during important periods of fetal development, which leads to potential harms, including congenital anomalies, preterm birth, and other adverse outcomes [18].

Studies from low- and middle-income countries reported the highest rates of SM practices [19]. For instance, SM prevalence in Ethiopia reached 44.6% among women during pregnancy, particularly for the treatment of nausea and headaches [20], whereas in Pakistan, a study reported that the prevalence of OTC medication among women during pregnancy reached 63.5% [21]. Comparably, in the Gulf region, studies conducted in countries such as Kuwait and the United Arab Emirates (UAE) reported moderate levels of SM, ranging from 30% to 50% [22,23]. According to the literature, there is a lack of empirical studies and reviews on SM among pregnant women in the context of Saudi Arabia. However, investigations in the general community found widespread use of SM without consulting healthcare professionals [24,25,26]. According to a recent study, 81.3% of pregnant women use SM without consultation [25].

Certain demographics and behaviors associated with SM were identified, including age, education level, past experience, occupation, and number of pregnancies [27,28,29]. However, cultural and family influences have an important impact [30]. In Saudi Arabia, SM is influenced by a variety of circumstances, including friends, family, and previous experience [26]. These SM behaviors may have serious consequences during pregnancy, including miscarriage, abnormalities, or developmental difficulties in the fetus [31,32]. Despite earlier studies on SM in Saudi Arabia’s general population, understanding the pattern, influence, and reasons for SM among pregnant women is considered a priority due to the repercussions of SM. In addition to identifying key variables associated with SM and awareness, which can help public health professionals design targeted educational campaigns and improve antenatal consultation strategies, this study aims to assess factors, attitudes, and prevalence of SM among pregnant women in Riyadh’s First Health Cluster.

## 2. Materials and Methods

### 2.1. Study Area/Setting

This study was conducted in the First Health Cluster of the Riyadh region, Saudi Arabia, encompassing urban, rural, and remote areas to capture a diverse representation of pregnant women across different geographic settings. This broad scope facilitates a comprehensive understanding of SM practices.

### 2.2. Study Subjects

The participants were pregnant women who were receiving prenatal care from various healthcare facilities. These women represent a diverse group from different socioeconomic backgrounds, educational levels, and geographical locations.

### 2.3. Inclusion Criteria

Pregnant women aged 18 years and above, residing in the Riyadh region, willing to participate in this study, and able to provide informed consent were eligible for inclusion. Additionally, participants had to be receiving prenatal care during the study period.

### 2.4. Exclusion Criteria

Pregnant women with a history of complicated medical conditions requiring specialized care, those with language or communication barriers, and individuals unwilling to participate in this study were excluded.

### 2.5. Study Design

This research adopts a cross-sectional study design to assess the prevalence of SM among pregnant women in Riyadh. Data were collected to provide a snapshot of SM practices within this specific population.

### 2.6. Sample Size

The sample size for this study was determined using a G-power analysis with an effect size of 0.2 from previous studies [11], a power of 0.8, and an alpha error probability of 0.05, resulting in a minimum sample size required of 148 [33]. However, to improve the precision of our study, we increased the sample size to 400 samples to represent the population, as recommended in the literature [34].

### 2.7. Sampling Technique

First, a stratified multistage sampling technique was utilized to ensure representation from different regions and healthcare settings, improving the generalizability of this study findings by categorizing the sample into three levels (primary, secondary, and tertiary). Within each stratum, a systematic sampling technique was used to collect data from the waiting area of prenatal clinics through sampling intervals by selecting every 4th patient. Due to the lack of official number of pregnant women distribution data across the healthcare level, an equal distribution approach was allocated (approximately 133–134 per level) in order to minimize potential bias from unknown population proportions.

### 2.8. Data Collection Methods, Instrument Used, and Measurements

The questionnaire was adapted based on previous investigations [10]. As recommended in the literature, the opinions and experiences of target participants, gathered through focus groups and interviews with experts in the field, were used to identify the items [35]. This was followed by amending and translating the questionnaire into the Arabic language, following the World Health Organization (WHO) recommendations for tool translation [36], which involve forward and backward translation into English to ensure the precision of the question’s meaning. A pilot study was conducted to assess the reliability and face validity among 5% of the sample before the commencement of the actual data collection, and amendments were made to the questionnaire based on the findings. Cronbach’s alpha was used to assess this study’s reliability, and the result was 0.89. In addition, content validity was ensured by a panel of physicians and public health professors to provide modifications and changes as appropriate to the instrument. This tool includes questions regarding SM practices, types of medications used, reasons for SM, and sources of information on medications (Appendix A). Key independent variables in this research include demographic characteristics of pregnant women (such as education level and socioeconomic status). Furthermore, in all instances where household income was mentioned, the currency was specified as Saudi Riyal (SAR) to ensure clarity and standardization. The dependent variables in this study are a history of chronic illnesses or medication use, access to healthcare services, and cultural beliefs influencing SM practices during pregnancy. The data collection was held from December 2024 to February 2025.

### 2.9. Statistical Analysis

SPSS version 25 was used for quantitative analysis and data management, including the coding of the raw data. This study involved calculating univariate analysis (frequency and percentages), while descriptive statistics were used in the analysis of the types of medications commonly self-administered, the reasons for SM, and the sources of information influencing medication choices during pregnancy. Furthermore, the bivariate analysis was conducted using chi-square tests to explore the associations between demographic variables and SM behaviors. Moreover, we executed a binary logistic regression analysis with SM as the dependent variable, while the independent variables in this model included nationality, age, household income, employment status, pregnancy trimester, and history of miscarriage. Furthermore, we estimated both unadjusted odds ratios (ORs) and adjusted odds ratios (AORs) for SM in the baseline model, while model fit was assessed using the Hosmer–Lemeshow goodness-of-fit test, overall classification accuracy, and Nagelkerke R^2^. Additionally, confidence intervals of 95% were calculated to evaluate the direction and strength of the relationship, with statistical significance set at *p* < 0.05. The data were visualized and presented in tables and figures.

### 2.10. Ethical Considerations

This study was carried out in compliance with the Declaration of Helsinki and approved by King Saud Medical City (KSMC) Institutional Review Board (or Ethics Committee) (H1R1-20-Nov24-04). All study participants provided informed consent, and the data acquired from the participants were kept confidential. The researcher did his utmost to maintain the anonymity and confidentiality of the participants’ information.

## 3. Results

### 3.1. Participant Sociodemographic and Obstetric Characteristics Related to Self-Medication

Among the 400 pregnant women, the majority were between 26 and 35 (58.8%), followed by the age group of 36 to 45 (35.8%), and only 5.5% of the participants were under the age of 25. Furthermore, 48.5% reported being in the middle-income group (9500–20,000), while 46.0% of participants were in the lower-income group, earning less than 9500 SAR, and only 5.5% of the participants reported earning more than 20,000 SAR. The high-income group was only represented by 5.5%, earning above 20,000. Regarding employment status, a substantial proportion (77.0%) were not working, while 15.3% were employed full-time, 5.3% part-time, and only 2.5% were students. The vast majority of participants were Saudi nationals (87.5%), with non-Saudis representing 12.5% of the sample. Most women (69.5%) were in their second trimester (14–27 weeks), followed by 16.8% in the third trimester and 13.8% in the first. Concerning obstetric history, 84.8% were multigravidae (had been pregnant more than once), while 15.3% were primigravidae (pregnant for the first time). In terms of miscarriage history, 45.8% reported no previous miscarriage, 33.3% had experienced one, 15.5% had experienced two, and 5.5% had experienced three miscarriages. Notably, the prevalence of SM among pregnant women is reported to be 36.5%.

Table 1 shows the analysis of SM practice, which revealed a significant association between several socio-demographic statuses and obstetric variables. For instance, age was associated with SM, with no women under 25 reporting SM (0.0%). However, 33.2% of women aged between 26 and 35 and 33.6% of those aged between 36 and 45 reported SM. Variables such as education were associated with SM (*p* < 0.05). The highest rate of SM was observed among women with no formal education (100.0%), followed by those with elementary education level (51.4%), while a lower rate was reported among those with high school (24.5%) and university education (26.3%). In terms of household income, there was no association with SM. However, higher occurrences of SM were observed among women in the upper-income group (50%) in comparison to those in the lower-income group (31.0%) and the middle-income group (29.9%).

In terms of occupation, there was an association between the use and occupation (*p*-value < 0.05). SM was high among pregnant women with part-time jobs (52.4%) compared to those with full-time jobs (18.0%). Despite that, nationality was not associated with SM (*p* = 0.081); a higher proportion of Saudi pregnant women practiced SM (32.9%) compared to non-Saudi women (22.0%). In terms of trimester, there was no association with SM (*p* = 0.581). However, there was an association between the number of pregnancies (*p* = 0.000), with 37.2% of multigravidae reporting SM. Nonetheless, miscarriage was found to be associated with SM (*p* < 0.05). Pregnant women who had experienced two miscarriages had the highest rate (62.9%), followed by those with only one miscarriage (31.6%), and those with no history of miscarriage (24.6%). Notably, these findings indicate that lower education, a history of miscarriage, increasing age, working part-time, and previous pregnancies are associated with a high rate of SM.

### 3.2. Awareness and Knowledge of Self-Medication

Table 2 shows participants’ knowledge and perceptions regarding antibiotic use and resistance. The majority of respondents (85.8%) correctly indicated that antibiotics cannot be obtained from a pharmacy without a doctor’s prescription. Nonetheless, 5.8% believed they could, and 8.5% were unsure. Most of the participants recognized that antibiotic resistance is a serious public health issue (48.0%), while 19.3% believed that it was not, and 32.8% reported uncertainty. Regarding viral infection, 45.3% incorrectly believed that antibiotics could treat viral infection. However, 37.0% answered correctly, and 17.8% were unsure. In terms of medication pricing and effectiveness, 52.0% disagreed with the idea that a higher price indicates a better product. However, 39.3% believed it did, and 8.8% were uncertain. Furthermore, only 31.0% agreed that newer antibiotics are more effective, 17% disagreed, and more than half of the participants (52.0%) expressed uncertainty. These findings illustrate major gaps in knowledge regarding appropriate antibiotic use and resistance, as well as prevalent misconceptions about medication efficacy and pricing.

### 3.3. Self-Medication Patterns

According to their reported health conditions, SM practices among pregnant women most commonly involved medications for hair loss, reported by 137 participants (29.91%), followed by weight loss (115 participants, 25.11%), menstrual problems (76 participants, 16.5%), and weight gain (63 participants, 13.76%). However, a few of the participants reported SM for blood pressure (43 participants, 9.39%), arthritis (13 participants, 2.84%), and diabetes mellitus (11 participants, 2.40%). In terms of medication type, antipyretic medications are the most prevalent (359 participants, 57.53%), followed by cough syrups (144 participants, 23.08%), antiallergic (59 participants, 9.46%), and antacid (51 participants, 8.17%), while drugs for diarrhea and constipation were the lease reported among the participants (11 participants, 1.76%).

Table 3 shows that the major reasons for non-use across various characteristics were statistically significant (*p*-value < 0.05). The women aged between 26 and 35 frequently reported fear of dangerous medication (87.5%), compared to those aged 36–45, who reporting concerns over improper medication use (53.0%) and wrong diagnoses (52.5%). Notably, pregnant women under the age of 25 only reported concern regarding negative drug effects (3.5%). Multigravida women reported concerns across all domains, particularly improper use (100%) and addiction risk (100%). On the other hand, primigravidae reported concern about negative drug effects (12.9%) and showed no concerns related to addiction. Similarly, analysis by miscarriage history showed that women with no history reported concerns about negative drug effects (48.9%) and inadequate medication knowledge (60.2%). In contrast, those with two miscarriages reported high concern about improper use (28.0%) and addiction (31.8%). Nonetheless, trimester was associated with differences in women’s concerns; women in the second trimester most frequently reported fear of dangerous medication (84.7%), followed by fear of improper use (59.1%) and addiction concerns (44.3%). These findings show that pregnancy-related experiences significantly shape perceptions and concerns about SM.

Table 4 shows that the use of medications by pregnant women for common health conditions, including fever, common cold, urinary tract infections (UTIs), pain and cramps, and sore throat, is associated with pregnant women’s characteristics (*p* value < 0.05). Age appeared to influence medication use; women aged between 26 and 35 reported the highest use of medication for fever (48.4%), sore throat (47.9%), common cold (57.6%), UTIs (100%), and runny nose (45.5%). Women aged between 36 and 45 had higher medication use for pain and cramps (91.7%). However, the age group under 25 showed minimal medication use, except for fever and the common cold. Nonetheless, pregnancy history also influenced medication use. Multigravidae were the primary users of medication across all conditions: 92.2% for fever, 90.7% for sore throat, and 100% for UTIs and pain. Women with no miscarriage history reported the highest medication use (50.8%): 51.2% for sore throat and 100% for pain. Moreover, the medications used were prevalent in the second trimester. These pregnant women accounted for the highest proportion of use in terms of fever (67.8%), sore throat (72.6%), common cold (67.6%), UTIs (66.7%), and runny nose (65.7%). However, pain and cramps were reported as high in the third trimester (91.7%). These findings show that medication use among pregnant women varies based on age, pregnancy experience, and miscarriage history. Women in their mid-30s, multigravidae, and those in the second or third trimesters were more likely to practice SM.

**Figure 1:**
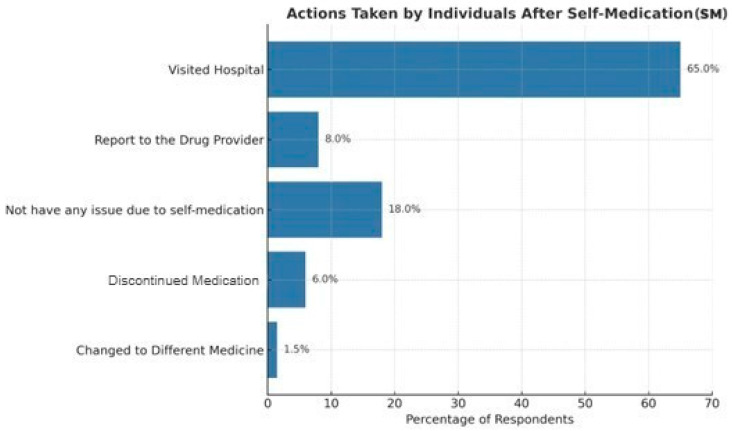
Response to drug-related issues.

Figure 1 shows that the resolution of post-medication issues included visiting hospitals (295 participants, 58.60%), having no issues with SM (68 participants, 15.18%), reporting to the drug provider (37 participants, 8.50%), discontinuing medication (14 participants, 3.18%), and changing to different medicines (9 participants, 2.09%).

### 3.4. Sources of Information for Self-Medication

**Figure 2:**
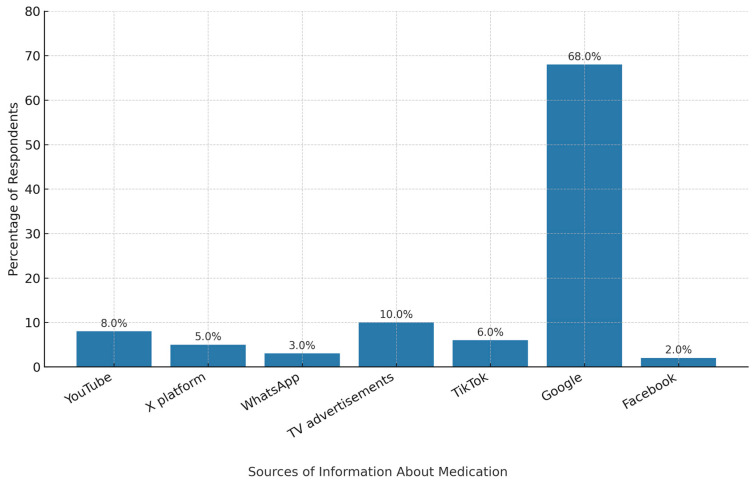
Participants’ electronic sources of information for self-medication.

The results show that the majority of participants’ source of information was personal experience (28.05%), followed by advice from social media (22.56%), pharmacists (15.55%), and family members (14.63%). Other sources include suggestions from friends (10.82%) and reuse of old prescriptions (8.38%). Figure 2 shows the sources of information, including social media and search engines, with Google being the most prevalent (286 participants, 66.05%), followed by TV advertisements (44 participants, 10.15%), YouTube (32 participants, 7.39%), and TikTok (24 participants, 5.54%) × platform (20 participants, 4.60%), WhatsApp (10, 2.30%), and Facebook.

### 3.5. Binary Logistic Regression Analysis

In the baseline regression model, the odds of SM were estimated to be 2.18 times higher compared to non-SM (Exp(B) = 2.175, *p* < 0.001). This suggests that without considering any predictors, there was a higher rate of SM in the sample. Nonetheless, this model explained almost 12% of the variance (Nagelkerke R^2^ = 0.118). However, the Hosmer–Lemeshow goodness-of-fit test indicated poor model fit (χ^2^(6) = 109.198, *p* < 0.001), and the classification table showed that the overall model correctly classified 66.5% of cases. Furthermore, Table 5 shows the regression model, which revealed that increasing age was significantly associated with a decrease in the odds of SM (OR = 0.919, 95% CI (confidence interval): 0.879–0.961, *p* < 0.001). Nonetheless, the higher-income variable was significantly associated with increased odds of SM. Women in the first and second higher-income categories had 2.86 times (*p* = 0.049) and 3.74 times (*p* = 0.009) higher odds of SM. Nationality also showed a significant association, with non-Saudi women having lower odds of SM (OR = 0.306, *p* = 0.029). The women with a history of miscarriage were 2.43 times more likely to practice SM (*p* < 0.001), whereas working status and current trimester were not significant. In terms of the AORs for the factors influencing SM, age was significantly associated with SM (*p*-value = 0.000), with an AOR of 0.928 (95% CI: 0.890–0.967), while working conditions were not significantly associated with SM, with a *p*-value of 0.308 and an AOR of 0.745 (95% CI: 0.439–1.297). The AOR for income level was 2.228 (95% CI: 0.913–5.438), with a *p*-value of 0.078, indicating that women in this category had approximately 2.2 times the odds of engaging in SM compared to the baseline income group.

## 4. Discussion

This study investigated the prevalence and determinants of SM. The findings revealed that a substantial proportion of pregnant women practice SM, with notable differences based on socioeconomic and obstetric characteristics. Almost a third of pregnant women practice SM, with the most common conditions leading to SM involving hair loss and weight loss. These findings align with previous investigations, which have shown that in Nigeria, a high level of SM was reported, particularly for hair loss [37]. However, our study is distinguished by its lower prevalence compared to South Asian regions, where over 60% of pregnant women engage in SM [38]. Notably, in Saudi Arabia, SM practices were highly prevalent among the general population compared to pregnant women [26,39].

Our results indicate that age is associated with SM, with women aged 26–45 years more likely to experience SM. This pattern is consistent with the study conducted in Turkey and Iran, which found that women during their reproductive years are more confident in using medication without medical advice [40,41,42,43]. Additionally, education level and occupation were found to influence and be associated with SM, suggesting a possible lack of awareness of SM risks. These findings contradict with a previous study conducted in Pakistan, in which more educated pregnant women were more likely to practice SM [44]. Interestingly, full-time employees were less likely to practice SM. Additionally, multigravidae were significantly more likely to practice SM compared to primigravidae. This could be due to a higher level of confidence from prior pregnancy experience.

In terms of awareness, participants’ knowledge regarding antibiotics was inadequate, despite their awareness of prescription requirements. For instance, nearly half of the participants held the misconception that antibiotics can treat viral infections. This misconception is consistent with global trends and major public health concerns, as well as the risk of antimicrobial resistance (AMR) [45]. Nonetheless, almost half recognized AMR as a serious health threat, underlining the need for educational interventions [46].

The majority of pregnant women feared developing drug addiction, as they were concerned about improper use and misdiagnosis. These findings reinforce the need to promote awareness. Despite these concerns, the most common sources included previous experience, advice from family, and pharmacists. These sources of information are consistent with the studies conducted in Jordan and Egypt [47,48]. SM usage varied among the pregnant women in this study. For instance, common cold, fever, and sore throat were among the major reasons for medication.

In terms of conditions treated with SM among pregnant women, the most commonly used medications were for fever, hair loss, weight management, menstrual problems, and common minor ailments. The category of medications used included multivitamins and nutritional supplements for hair and weight concerns, antipyretics and analgesics for fever and pain, antacids for gastrointestinal discomfort, and herbal remedies. Notably, according to the Food and Drug Administration (FDA) pregnancy risk classification, most antipyretics and commonly used analgesics fall under category B, indicating no evidence of risk in humans when used appropriately. However, certain herbal remedies could fall under categories C or D, given the potential teratogenicity or limited safety data [49].

Notably, some pregnant women reported using multivitamins or nutritional supplements without medical consultation or professional instruction, which may result in exceeding the recommended dosage of vitamins such as vitamin A, potentially leading to unintended weight changes or teratogenic effects [50,51]. Moreover, certain medications, such as Ibuprofen, which is classified as FDA Category B during the first and second trimesters of pregnancy, indicate no evidence of risk in humans when used appropriately. However, this medication during the third trimester is considered FDA Category D, given the potential risks of premature closure of the ductus arteriosus or fetal kidney dysfunction, which can lead to oligohydramnios and neonatal renal failure [52]. Therefore, understanding the risk profile of these medications is an important task for the public health professionals in antenatal counseling and for policy makers to minimize risks and fetal harm.

In terms of post-medication issues, pregnant women tend to seek help from hospitals. However, a minority either informed the drug provider, switched to another drug, experienced no complications, or discontinued the medication. This study’s results are consistent with previous findings by Al-Qahtani, Alqahtani [53], which reported that most individuals who practiced SM did so with precautions, such as consistently following package instructions, in addition to avoiding sharing medications with others. Regarding sources of SM information, personal experience and advice from social media were most influential, followed by guidance from pharmacists, family members, friends, or reusing old prescriptions. Similarly, a previous investigation by Alghadeer, Aljuaydi [54] noted that online platforms frequently shaped SM attitudes, with pharmacists and family members playing a secondary role.

Although the logistic regression model demonstrated weak explanatory power (Nagelkerke R^2^ = 0.071) and a statistically significant Hosmer–Lemeshow test (*p* < 0.001), it provided valuable insights into the predictors of SM. Age, household income, and miscarriage history emerged as significant predictors, while working status, nationality, social media, and current trimester were not significant. The model was more accurate in predicting non-self-medicators than self-medicators, as reflected in the overall classification accuracy of 65.8%.

In addition to the socio-demographic factors, our results on minor illnesses and drug categories provide important insight into the risk classification. The most common reasons for SM were headache, nausea, and mild respiratory symptoms. Nonetheless, antipyretics and analgesics are reported to be the most frequently used medication categories, which are considered lower risk when used appropriately among pregnant women. However, certain medications, such as antibiotics and herbal substances, pose greater risks due to potential teratogenic effects, unknown safety data, and drug interactions. Therefore, understanding these trends and patterns can inform educational efforts by emphasizing which conditions and concerns warrant professional education and physician oversight, as well as identifying which medications are unsafe. Therefore, public health initiatives can integrate these reported results into actionable plans towards targeted pharmacy-based counseling and awareness campaigns in order to discourage unsupervised SM, particularly for the use of high-risk drug categories during pregnancy.

Future public health campaigns, particularly during antenatal visits, should include actionable interventions, and healthcare providers should improve screening for SM during antenatal visits and provide appropriate counseling, particularly regarding fetal safety. Nonetheless, future research needs to examine the causal association between SM and adverse pregnancy outcomes through rigorous methods such as longitudinal or case–control studies, as well as explore the influence of pharmacist counseling, cultural norms, and online platforms as sources of information on SM behaviors.

These important implications for both clinical practice and health policy can improve antenatal care services by incorporating routine screenings to address SM attitudes, particularly among women with lower education and multigravidae, in addition to providing counseling on the risks associated with unsupervised drug use. Furthermore, targeted training programs for clinicians and pharmacists can improve their ability to raise awareness among pregnant women on the proper use of medication and when to seek medical advice. Moreover, healthcare services need to strengthen access to antenatal consultations and primary care in order to ensure that pregnant women do not rely on OTC medications for convenience. Nonetheless, stricter regulation of OTC medication and community-based awareness can reduce the harmful attitude, given that these strategies can play a critical role in informing the population and managing risk patterns. As a result, they help to prevent adverse maternal outcomes and promote a healthy fetus.

Certain limitations in this study need to be acknowledged. First, potential sampling bias may have influenced the results of this study. Secondly, language and literacy barriers could affect the accuracy of data, particularly among non-Saudi participants. Thirdly, the lack of clinical verification of SM practices and their outcome without confirmation of the type and/or dosage or frequency of drug use may affect the accuracy of this study, which also relies only on self-reported data, which could also result in recall bias, misreporting, and social desirability. In addition, this study’s limitation is revealed in the restriction to one health cluster, which may not reflect the behaviors of women across different regions, as well as the research design, which cannot establish causality among these variables.

Despite these limitations, this study is one of the few studies conducted in the Riyadh region specifically addressing SM among vulnerable individuals (pregnant women). This study provides in-depth details on a variety of characteristics and age groups, examining SM behaviors, awareness, and prevalence. Future studies should overcome these limitation through targeted and longitudinal designs to assess SM over time, incorporate community-based sampling, and ensure broader representation and more accurate assessments. In addition, future studies could incorporate clinical validation, prescription records, or pharmacy data to provide a more precise estimation of SM practices and their potential effects on maternal and fetal health.

## 5. Conclusions

This study found that one-third of the pregnant women in the Riyadh First Health Cluster practice SM with the most commonly used medications including antipyretics, cough syrups, and nutritional supplements for conditions such as weight management, hair loss, and minor ailments. Furthermore, socio-demographic and obstetric characteristics such as education level, age, part-time employment, and multigravida status, in addition to miscarriage history were significantly associated with SM. Nonetheless, misconceptions about antibiotic use and resistance, as well as reliance on informal sources of information regarding SM, such as social media and personal experience, highlight persistent awareness and knowledge gaps.

These findings highlight the urgent need for public health initiatives to promote and strengthen antenatal counseling, enhance community-based awareness, and enforce strict regulation of OTC medication. Addressing these determinants can reduce unsafe use of drugs, improve maternal and fetal health outcomes, and guide future educational and policy development.

## Figures and Tables

**Table 1:** Association between pregnant women’s characteristics and use of self-medication.

Pregnant Women Characteristic		Do You Self-Medicate?		
		Yes	No	
		N (%)	N (%)	*p*-value
Age				
	Under 25	0 (0.0)	22 (100.0)	0.000 *
	26–35	78 (33.2)	157 (66.8)	
	36–45	48 (33.6)	95 (66.4)	
Education level	Elementary school level	36 (51.4)	34 (48.6)	0.000 *
	High school level	45 (24.5)	139 (75.5)	
	University level	36 (26.3)	101 (73.7)	
	No education	9 (100.0)	0 (0.0)	
Household income (per month)				
	Lower class < 9500	57 (31.0)	127 (69.0)	0.154
	Middle class 9500–20,000	58 (29.9)	136 (70.1)	
	Upper class > 20,000	11 (50.0)	11 (50.0)	
Occupation				
	Student	0 (0.0)	10 (100.0)	0.002 *
	Not working	104 (33.8)	204 (66.2)	
	Working full-time	11 (18.0)	50 (82.0)	
	Working part-time	11 (52.4)	10 (47.6)	
	Retired	0 (0.0)	0 (0.0)	
Nationality				
	Saudi	115 (32.9)	235 (67.1)	0.081
	Non-Saudi	11 (22.0)	39 (78.0)	
Current trimester				
	First trimester (1–13 weeks)	14 (25.5)	41 (74.5)	0.581
	Second trimester (14–27 weeks)	90 (32.4)	188 (67.6)	
	Third trimester (28–41 weeks)	22 (32.8)	45 (67.2)	
Number of pregnancies				
	Primigravidae	0 (0.0)	61 (100.0	0.000 *
	Multigravidae	126 (37.2)	213 (62.8)	
Number of miscarriages				
	Did not happen	45 (24.6)	138 (75.4)	0.000 *
	Once	42 (31.6)	91 (68.4)	
	Twice	39 (62.9)	23 (37.1)	
	Three	0 (0.0)	22 (100.0)	

(*) = Significant *p*-value level at ≤0.05.

**Table 2:** Awareness of self-medication among pregnant women.

Question		N	%
Can I get antibiotics at the pharmacy without a prescription from a doctor?			
	Yes	23	5.8
	No	343	85.8
	I don’t know	34	8.5
	Total	400	100.0
Is antibiotic resistance a serious issue for public health in our country?			
	Yes	192	48.0
	No	77	19.3
	I don’t know	131	32.8
	Total	400	100.0
Can antibiotics be used to treat viral infections?			
	Yes	181	45.3
	No	148	37.0
	I don’t know	71	17.8
	Total	400	100.0
Does a higher price indicate a better product?			
	Yes	157	39.3
	No	208	52.0
	I don’t know	35	8.8
	Total	400	100.0
Are newer antibiotics more effective?			
	Yes	124	31.0
	No	68	17.0
	I don’t know	208	52.0
	Total	400	100.0

**Table 3:** Association between pregnant women’s characteristics and non-use of self-medication.

Characteristic		Non-Use of Self-Medication						
		Negative Drug Effects	Fear of Dangerous Medication	Concern over Improper Use	Fear of Wrong Diagnosis	Inadequate Medication Knowledge	Developing a Drug Addiction	
		N (%)	N (%)	N (%)	N (%)	N (%)	N (%)	*p*-Value
Age								
	Under 25	11 (3.5)	0 (0.0)	0 (0.0)	0 (0.0)	0 (0.0)	11 (12.5)	0.000 *
	26–35	177 (56.9)	63 (87.5)	62 (47.0)	57 (47.5)	42 (47.7)	14 (15.9)	
	36–45	123 (39.5)	9 (12.5)	70 (53.0)	63 (52.5)	46 (52.3)	63 (71.6)	
	46–55	0 (0.0)	0 (0.0)	0 (0.0)	0 (0.0)	0 (0.0)	0 (0.0)	
	56 and older	0 (0.0)	0 (0.0)	0 (0.0)	0 (0.0)	0 (0.0)	0 (0.0)	
Number of pregnancies								
	Primigravidae	40 (12.9)	9 (12.5)	0 (0.0)	11 (9.2)	10 (11.4)	0 (0.0)	0.000 *
	Multigravida	271 (87.1)	63 (87.5)	132 (100.0)	109 (90.8)	78 (88.6)	88 (100.0)	
Number of miscarriages								
	Did not happen	152 (48.9)	40 (55.6)	47 (35.6)	47 (39.2)	53 (60.2)	47 (53.4)	0.000 *
	Once	106 (34.1)	21 (29.2)	26 (19.7)	45 (37.5)	13 (14.8)	13 (14.8)	
	Twice	53 (17.0)	0 (0.0)	37 (28.0)	28 (23.3)	0 (0.0)	28 (31.8)	
	Three	0 (0.0)	11 (15.3)	22 (16.7)	0 (0.0)	22 (25.0)	0 (0.0)	
	More than three	0 (0.0)	0 (0.0)	0 (0.0)	0 (0.0)	0 (0.0)	0 (0.0)	
Current trimester	First trimester (1–13 weeks)	27 (8.7)	0 (0.0)	21 (15.9)	25 (20.8)	23 (26.1)	14 (15.9)	0.000 *
	Second trimester (14–27 weeks)	238 (76.5)	61 (84.7)	78 (59.1)	60 (50.0)	31 (35.2)	39 (44.3)	
	Third trimester (28–41 weeks)	46 (14.8)	11 (15.3)	33 (25.0)	35 (29.2)	34 (38.6)	35 (39.8)	

(*) = Significant *p*-value level at ≤0.05.

**Table 4:** Association between pregnant women’s characteristics and medication use.

Characteristic		Medication Use						
		Fever	Sore Throat	Common Cold	Urinary Tract Infection	Pains and Cramps	Runny Nose	
		N (%)	N (%)	N (%)	N (%)	N (%)	N (%)	*p*-Value
Age								
	Under 25	11 (4.3)	1 (0.5)	11 (3.6)	0 (0.0)	1 (8.3)	0 (0.0)	0.000 *
	26–35	125 (48.4)	103 (47.9)	178 (57.6)	30 (100.0)	0 (0.0)	65 (45.5)	
	36–45	122 (47.3)	111 (51.6)	120 (38.8)	0 (0.0)	11 (91.7)	78 (54.5)	
	46–55	0 (0.0)	0 (0.0)	0 (0.0)	0 (0.0)	0 (0.0)	0 (0.0)	
	56 and older	0 (0.0)	0 (0.0)	0 (0.0)	0 (0.0)	0 (0.0)	0 (0.0)	
Number of pregnancies								
	Primigravidae	20 (7.8)	20 (9.3)	50 (16.2)	0 (0.0)	0 (0.0)	20 (14.0)	0.000 *
	Multigravida	238 (92.2)	195 (90.7)	259 (83.8)	30 (100.0)	12 (100.0)	123 (86.0)	
Number of miscarriages								
	Did not happen	131 (50.8)	110 (51.2)	147 (47.6)	0 (0.0)	12 (100.0)	71 (49.7)	0.000 *
	Once	65 (25.2)	43 (20.0)	112 (36.2)	20 (66.7)	0 (0.0)	33 (23.1)	
	Twice	62 (24.0)	51 (23.7)	39 (12.6)	0 (0.0)	0 (0.0)	28 (19.6)	
	Three	0 (0.0)	11 (5.1)	11 (3.6)	10 (33.3)	0 (0.0)	11 (7.7)	
	More than three	0 (0.0)	0 (0.0)	0 (0.0)	0 (0.0)	0 (0.0)	0 (0.0)	
Current trimester	First trimester (1–13 weeks)	37 (14.3)	24 (11.2)	44 (14.2)	0 (0.0)	0 (0.0)	24 (16.8)	0.000
	Second trimester (14–27 weeks)	175 (67.8)	156 (72.6)	209 (67.6)	20 (66.7)	1 (8.3)	94 (65.7)	
	Third trimester (28–41 weeks)	46 (17.8)	35 (16.3)	56 (18.1)	10 (33.3)	11 (91.7)	25 (17.5)	

(*) = Significant *p*-value level at ≤0.05.

**Table 5:** Binary logistic regression model for the prediction of pregnant women’s self-medication.

Variables	*p*-Value	AOR (95%CI)	*p*-Value	COR (95%CI)
Age	0.000 *	0.928 [0.890–0.967]	0.000 *	0.919 [0.879–0.961]
Working status	0.308	0.745 [0.439–1.297]	0.174	1.803 [0.770–4.220]
Household income (Ref)				
Household income	0.078	2.228 [0.913–5.438]	0.049 *	2.862 [1.004–8.158]
Household income	0.061	2.345 [0.962–5.713]	0.009 *	3.735 [1.380–10.103]
Nationality	0.126	0.576 [2.850–1.167]	0.029 *	0.306 [0.105–0.886]
Current trimester (Ref)				
Current trimester (1)	0.375	1.432 [0.648–3.164]	0.875	1.075 [0.437–2.644]
Current trimester (2)	0.942	1.021 [0.578–1.803]	0.197	0.654 [0.343–1.247]
Miscarriage status	0.007 *	1.826 [1.183 2.821]	0.000 *	2.432 [1.482–3.991]

Cl = confidence level, AOR = adjusted odd ratio, COR = crude odds ratio, (*) = significant *p*-value level at ≤0.05.

## Data Availability

The data presented in this study are available on request from the corresponding author. The data are not publicly available because privacy access requires further requests and approval.

## Appendix A. Questionnaire

Self-Medication Practice	ممارسة العلاج الذاتي
Do you self-medicate? O Yes O No Can I get antibiotics at the pharmacy without a prescription from a doctor? O Yes O No O I don’t know Is antibiotic resistance a serious issue for public health in our country? (know) O Yes O No O I don’t know Can antibiotics be used to treat viral infections? O Yes O No O I don’t know Does a higher price indicate a better product? O Yes O No O I don’t know Are newer antibiotics more effective? O Yes O No O I don’t know	هل تستخدم العلاج الذاتي؟ O نعم O لا هل يمكنني الحصول على المضادات الحيوية من الصيدلية دون وصفة طبية من الطبيب؟ O نعم O لا O لا اعلم هل مقاومة المضادات الحيوية مشكلة خطيرة بالنسبة للصحة العامة في بلدنا؟ O نعم O لا O لا اعلم هل يمكن استخدام المضادات الحيوية لعلاج الالتهابات الفيروسية؟ O نعم O لا O لا اعلم هل السعر الأعلى يشير إلى منتج أفضل؟ O نعم O لا O لا اعلم هل المضادات الحيوية الأحدث أكثر فعالية؟ O نعم O لا O لا اعلم
Uses of self-medication O Common cold O Fever O Sore throat O Runny nose O Urinary tract infection O Pains and cramps O Diarrhea O Vomiting O Small wounds Commonly used drugs O Antipyretic O Antiallergic O Cough syrups O Antibiotics O Antacid (any type of digestive syrups) O Contraceptives O Diarrhea and constipation drugs	استخدامات العلاج الذاتي O زُكام O الحمى O التهاب الحلق O سيلان الأنف O التهاب المسالك البولية O آلام وتشنجات O الإسهال O القيء O على الجروح الصغيرة الأدوية شائعة الاستخدام Oخافض للحرارة Oمضاد الحساسية O شراب السعال O المضادات الحيوية O مضاد للحموضة O وسائل منع الحمل O أدوية الإسهال والإمساك
Information Source for Self-Medication	مصدر المعلومات العلاج الذاتي
Media sources for self-medication O TV advertisements O Newspapers O Magazines O Google O WhatsApp O YouTube O Instagram O X O tiktok Arguments against self-medication O Concern about negative drug effects O Concern about developing a drug addiction O Fear of a wrong diagnosis O Concern over the improper use of medications O Fear of dangerous medications O Inadequate medication knowledge O Others, if any Practicing self-medication for different ailments under the influence of advertisements O Weight loss O Diabetes O Hair loss O Wight gain O Sexual problems O Blood pressure O Menstrual problems O Arthritis People’s responses to drug-related issues brought on by self-medication O Report to drug provider O Visited hospital O Changed to a different medicine O Put an end to medication O Not have any issue due to self-medication Information sources O Previous experience O Old prescriptions O Suggested by pharmacist O Suggested by family members O Suggested by friends O Advertisements O From search engines (Google, etc.)	المصادر الإعلامية لأخذ الأدوية الذاتية O إعلانات تلفزيونية O الصحف O المجلات O جوجل (محركات البحث) O واتس اب O الفيسبوك O يوتيوب O انستغرام O اكس (تويتر) O تيك توك أسباب عدم أخذ الأدوية بدون وصفة طبية O القلق بشأن التأثيرات السلبية الدوائية O القلق من إدمان الأدوية O الخوف من التشخيص الخاطئ O القلق بشأن الاستخدام الغير صحيح للأدوية O الخوف من الأدوية الخطيرة O عدم المعرفة بشكل كافي للأدوية O أخرى إن وجدت ممارسة العلاج الذاتي للأمراض المختلفة تحت تأثير الإعلانات O فقدان الوزن O مرض السكري O تساقط الشعر O زيادة الوزن O مشاكل جنسية O ضغط الدم O مشاكل الدورة الشهرية O التهاب المفاصل استجابات الناس للمشاكل الناجمة من استخدام العلاج بدون وصفه O إبلاغ مزود الدواء O زيارة المستشفى O التغيير إلى دواء آخر O وضع حد للأدوية O ليس لديك أي مشكلة بسبب العلاج الذاتي مصادر المعلومات O الخبرة السابقة O الوصفات الطبية القديمة O يقترحها الصيدلي O يقترحها أفراد العائلة O يقترحها أصدقاء O الإعلانات O من محرك البحث (قوقل, الخ..)
